# Applications of Uncrewed Aerial Vehicles (UAV) Technology to Support Integrated Coastal Zone Management and the UN Sustainable Development Goals at the Coast

**DOI:** 10.1007/s12237-021-01001-5

**Published:** 2021-10-18

**Authors:** Sarah Kandrot, Samuel Hayes, Paul Holloway

**Affiliations:** 1 MaREI, the SFI Research Centre for Energy, Climate and Marine, Environmental Research Institute Beaufort Building, University College Cork, Haulbowline Road, Ringaskiddy, Co., Cork, P43 C573 Ireland; 2 Department of Geography, University College Cork, College Road, Cork, T12 K8AF Ireland; 3 Environmental Research Institute, University College Cork, Lee Road, Cork, T23 XE10 Ireland; 4 Green Rebel, Crosshaven Boat Yard, Point Road, Co., Cork, P43 EV21 Ireland

**Keywords:** Drones, Sustainability, Sustainable development goals, Integrated coastal zone management

## Abstract

Data and information obtained from low-cost uncrewed aerial vehicles (UAVs), commonly referred to as ‘drones’, can be used to support integrated coastal zone management (ICZM) and sustainable development at the coast. Several recent studies in various disciplines, including ecology, engineering, and several branches of physical and human geography, describe the applications of UAV technology with practical coastal management potential, yet the extent to which such data can contribute to these activities remains underexplored. The main objective of this paper is to collate this knowledge to highlight the areas in which UAV technology can contribute to ICZM and can influence the achievement of the UN Sustainable Development Goals (SDGs) at the coast. We focus on applications with practical potential for coastal management activities and assess their accessibility in terms of cost, ease of use, and maturity. We identified ten (out of the 17) SDGs to which UAVs can contribute data and information. Examples of applications include surveillance of illegal fishing and aquaculture activities, seaweed resource assessments, cost-estimation of post-storm damages, and documentation of natural and cultural heritage sites under threat from, for example, erosion and sea-level rise. An awareness of how UAVs can contribute to ICZM, as well as the limitations of the technology, can help coastal practitioners to evaluate their options for future management activities.

**Supplementary Information:**

The online version contains supplementary material available at 10.1007/s12237-021-01001-5.

## Introduction

Coastal zones are at the frontline of sustainability challenges arising from the exploitation of natural resources. Integrated coastal zone management (ICZM) seeks to address these challenges by balancing multiple, and sometimes conflicting, objectives within the limits set by natural dynamics. To do so effectively requires a reliable evidence-base upon which decision makers, such as policymakers and planners, can make informed management decisions. Uncrewed aerial vehicle (UAV) technology is well placed to support ICZM activities because it can deliver relevant data and information for evidence-based decision making.

Sustainability is at the heart of ICZM. The challenge for society is how to balance economic growth with environmental, social, cultural, and recreational objectives, all while ensuring we can meet “the needs of the present without compromising the ability of future generations to meet their own needs” (World Commission on Environment and Development 1987, p. 16). This is especially challenging at the coast, where populations and industries tend to be concentrated within or adjacent to sensitive ecosystems. Sustainability challenges are recognised in international policy agendas, notably in the 2030 Agenda for Sustainable Development, which sets out the sustainable development goals (SDGs) (UN General Assembly 2015). The SDGs are composed of 17 goals that address global challenges related to poverty, inequality, climate change, environmental degradation, peace, and justice. For each goal, a number of targets have been defined, and for (most of) the targets, there are a number of indicators and metrics for measuring progress towards those targets, and, ultimately, the overall goal.

Complementing the SDGs are other global initiatives, including the Convention on Biological Diversity (CBD) (United Nations 1992), the Sendai Framework for Disaster Risk Reduction (United Nations 2015a), and the Conference of the Parties (COP21) Paris Agreement (United Nations 2015b). In recent years, satellite-based Earth Observation (EO) products have become more accessible and widely used to address coastal and marine management issues, with opportunities to support the SDGs and other initiatives highlighted by Politi et al. (2019). The data requirements to deliver progress towards these conventions are substantial (Politi et al. 2019), but UAV technology can play a role in helping to deliver those requirements (Kitonsa and Kruglikov 2018). However, such opportunities offered by UAV platforms are yet to be fully explored or exploited compared to traditional EO products.

In this paper, we provide a brief overview of UAV and related technology. Next, we present an integrative review of the applications of UAV technology to support ICZM and the UN SDGs at the coast. For case studies that provided sufficient detail, we assessed their use of UAV technology based on 3 criteria: cost, ease of use, and maturity. This was performed using a 4-point scale for each criterion, which was then combined for an overall “accessibility” criterion (Table 1). We then assessed the potential for UAV technology to contribute to the UN SDGs at the coast by cross-referencing relevant goals and indicators with the applications identified from the review. Some of the applications described were not necessarily coastal but could be applied in a coastal setting. To conclude, we perform an intercomparison between UAV applications, their accessibility, and SDG goals, followed by a discussion on the challenges and future research requirements in relation to coastal applications of UAV technology.

**Table 1:** Criteria and descriptions used in assessing the cost, ease of use, and maturity of UAV applications

Rating	Cost	Description	Ease of use	Description	Maturity	Description
4	Low	< €2000	Very high	Simple off-the-shelf equipment for visual observation and simple tasks	Very high	Simple and commonly used tasks/equipment and a proven track record
3	Medium	€2–5000	High	Some processing of imagery and advanced analysis but common software. Some more advanced sensors	High	Somewhat common, though less widespread with some mixed results
2	High	€5–20,000	Medium	Advanced image processing and use of a variety of different sensors. Larger more complex drones with custom elements	Medium	Partly experimental, but similar methods/equipment previously used in related projects
1	Very high	> €20,000	Low	Largely custom built or advanced equipment. Use of machine learning, AI and other advanced processing methods	Low	Mostly experimental/proof of concept for analysis and equipment

## UAV and Related Technology

### UAV Platforms

UAV platforms can be split into two broad categories: Rotary and fixed wing. While there are some other platforms that contain elements from both categories and additional features, multi-rotor and fixed-wing platforms tend to dominate the market, each having pros and cons relative to the other. Rotary systems (typically multi-rotors) consist of one or more motors with spinning blades to provide lift, like a helicopter. Multi-rotors have the ability to hover, providing more precise control over the UAV positioning. Multi-rotor platforms tend to offer some of the most affordable and user-friendly options. For example the DJI phantom series cost under €2000, and these off-the-shelf, consumer-grade UAVs are capable of greater than 4 K video recording and 20MP photos on a 3-axis gimbal, in-built satellite and vision positioning with obstacle avoidance, a flight time of up to 30 min, and flight planning capabilities for consistent repeat surveys, to name some features. As the multi-rotor prices increase, so too do their capabilities. For example, the enterprise-level DJI Matrice 300 RTK (~ €12,000: dji.com/uk/matrice-300) contains the capabilities inherent to the cheaper multi-rotors, plus extra features such as the ability to carry a payload of multiple sensors up to 2.7 kg in weight (such as professional grade cameras or multi-spectral sensors), operate in a wider range of environmental conditions, with centimetre scale positional accuracy, more advanced onboard AI and automated functions, and a flight time of up to 55 min.

On the other hand, fixed-wing systems act more like an aeroplane, generating lift from its wings at speed. These platforms tend to be more expensive than multi-rotor UAVs, but are sometimes preferred for aerial mapping applications due to their long flight times and ability to cover large areas. An example is the SenseFly Ebee (sensefly.com/drones/) that can remain airborne for 90 min, maintain a cruising speed of up to 110 km/h, survey up to 500 ha in one flight, and provide cm scale positional accuracy, with a price of over €20,000 for the basic model. Since many fixed-wing UAVs use petrol engines as their power source, they can stay in flight longer than battery-operated UAVs. For example, the Penguin C (uavfactory.com/en/penguin-c-uas) can fly for up to 20 h and carry a payload of 2 kg. With multi-hour flight times and ability to carry large payloads, the fixed-wing UAV costs can sometimes run into six figures. Furthermore, some small fixed-wing models can be launched by hand, but larger models require a runway or catapult to launch. Because of their design, fixed-wing UAVs usually require more experience to fly than multi-rotor UAVs.

### Payloads

Many UAVs can be fitted with a variety of active and passive sensors, as well as mechanical payloads, allowing for a huge variety of applications, many of which are described later in this article. The majority of basic consumer-grade UAVs come with an integrated RGB sensor as standard. RGB meaning it captures light in the Red, Green, and Blue portions of the electro-magnetic spectrum (EMS), generating visible imagery much like consumer-grade handheld digital cameras. Images captured from these sensors can be used in many applications, from tourist videos (Osanna 2020) to photogrammetry (Scarelli et al. 2017). However, other applications require sensors that measure different portions of the EMS, apart from or in addition to the visible RGB portion. As these sensors can capture information not visible to the naked eye, they can provide information suitable for a variety of additional tasks, for example, detecting variations in plant health and biomass across a survey area (Wich and Koh 2018), or monitoring water quality (Kislik et al. 2018).

Thermal infra-red cameras are also commonly used on UAV platforms. For cheaper versions, they can measure relative temperature differences, but more advanced radiometric sensors can measure absolute temperatures. Their uses range from infrastructure inspections to fire fighting and search and rescue activities (Burke et al. ﻿2019).

Many UAVs are now designed for or capable of carrying LiDAR devices for capturing highly accurate surface elevation measurements and some shallow water ground elevation measurements. LiDAR works by emitting laser pulses from the instrument and then measuring the time and intensity of the return signal, providing information on the elevation (distance) and properties of the ground surface. Mechanical payloads are also becoming more common. These include the ability to drop off items, such as a floatation device (Seguin et al. 2018), or to collect physical samples, such as blow samples from whales (Geoghegan et al. 2018).

Finally, the larger and often more expensive UAVs are capable of carrying payloads of several kilograms, meaning they can manage an array of different sensors, or single large sensors. Conversely, the smaller, cheaper UAVs will have limited or no capability of carrying extra or different sensors. Additionally, the sensors, beyond the standard RGB versions, often carry additional costs. For example, the MicaSense RedEdge camera can detect two additional wavelengths beyond the standard RGB (micasense.com/rededge-mx/) with a cost of over €4000, while the DJI Zenmuse L1 LiDAR (dji.com/uk/zenmuse-l1) device costs approximately €15,000.

### Software and Additional Equipment

Most UAVs will come with controllers and/or software for smart phones, tablets, or laptops. These can be used for flight controlling, camera operations, and a variety of other functions. For some simple photo or video capturing applications, like creating virtual tourism videos, or visual infrastructure surveys, no additional apparatus may be required. However, with additional accuracy requirements, processing or advanced classification, further software, equipment, and training may be necessary.

For example, in order to perform high-accuracy repeat photogrammetric surveys on a site, several additional pieces of equipment and software need to be included. Flight programming software will allow you to pre-determine your flight route and save the coordinates for near identical repeat surveys. Ground control points will be necessary to accurately geolocate your imagery i.e. place your images in real-world coordinates. This requires surveying stable, clear objects throughout the survey area, or distributing markers to survey instead. Depending on the accuracy requirements, professional grade survey equipment or UAVs will be needed, adding anywhere from €5000 to over €20,000 in costs. Finally, photogrammetric software is required to turn the UAV images into digital surface models. Software and training costs will vary based on the licensing arrangements and pre-existing expertise. For many applications, further training and equipment will be required to maximise UAV potential and ensure accurate and reliable data collection and analysis.

## Applications of UAV Technology to Support Sustainable Development and ICZM

**Fig. 1:**
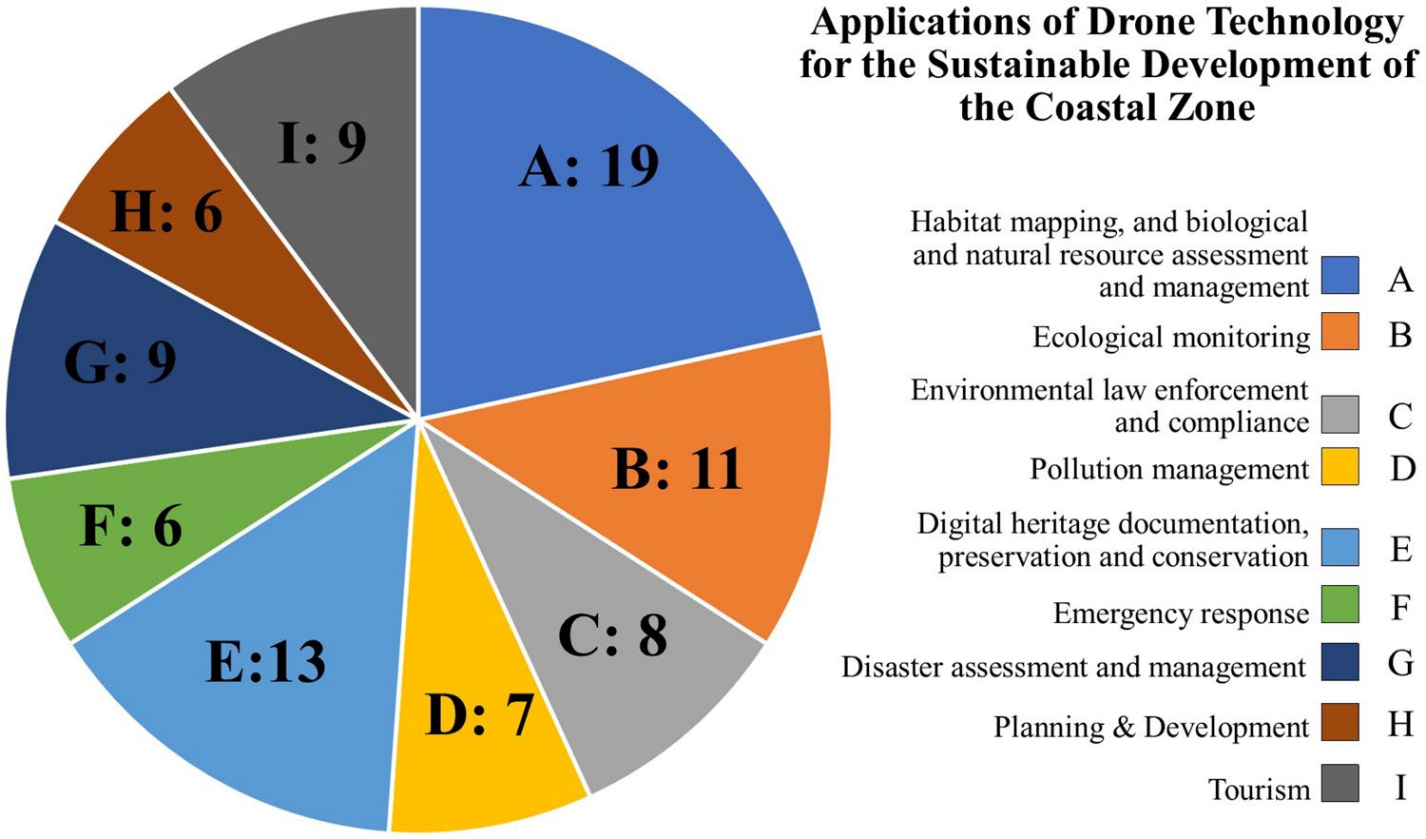
Nine areas in which UAV technologies can support ICZM and the UN SDGs at the coast and the number of references associated with each application

We searched both the academic and grey literature for examples documenting the use of UAVs for ICZM and/or sustainable development. We chose to include grey literature in our analysis due to the extensive usage of the technology outside of academia (e.g. by private consultancies and government agencies). In addition, the lag in academic publications, taking into account the speed at which the technology is advancing, limits our ability to appreciate fully the current extent of UAV employment for coastal applications. A search of electronic databases, including Google, Google Scholar, and the 278 databases contained in the University College Cork Library, was conducted using the keywords “drone”, “UAV”, “SUA”, “coast”, “ICZM”, “SDGs”, and “sustainable development”. References were selected on the basis of their relevance and age, with work published in the last 3 years prioritised, but older work, where relevant, not excluded. We selected and reviewed almost 100 references and categorised the applications into nine areas (Fig. 1; Supplementary Information 1).

### Habitat Mapping, and Biological and Natural Resource Assessment and Management

The use of UAVs for habitat mapping and biological and natural resource assessments are among the most common applications of UAV technologies in the coastal zone (Fig. 1). Information from such surveys is widely used for sustainable management and conservation purposes (Wich and Koh 2018). UAVs can help to map key habitats and to monitor ecological populations, behaviour, and health (Ventura et al. 2018; Pirotta et al. 2017; Lee et al. 2019). They can also be used to manage human interactions with marine species and to monitor other anthropogenic activities that can have negative environmental impacts (Femmami 2019). This information is important for protecting threatened marine life and for supporting Blue Growth, by providing policymakers, planners, and managers with an evidence-base for decision making.

**Fig. 2:**
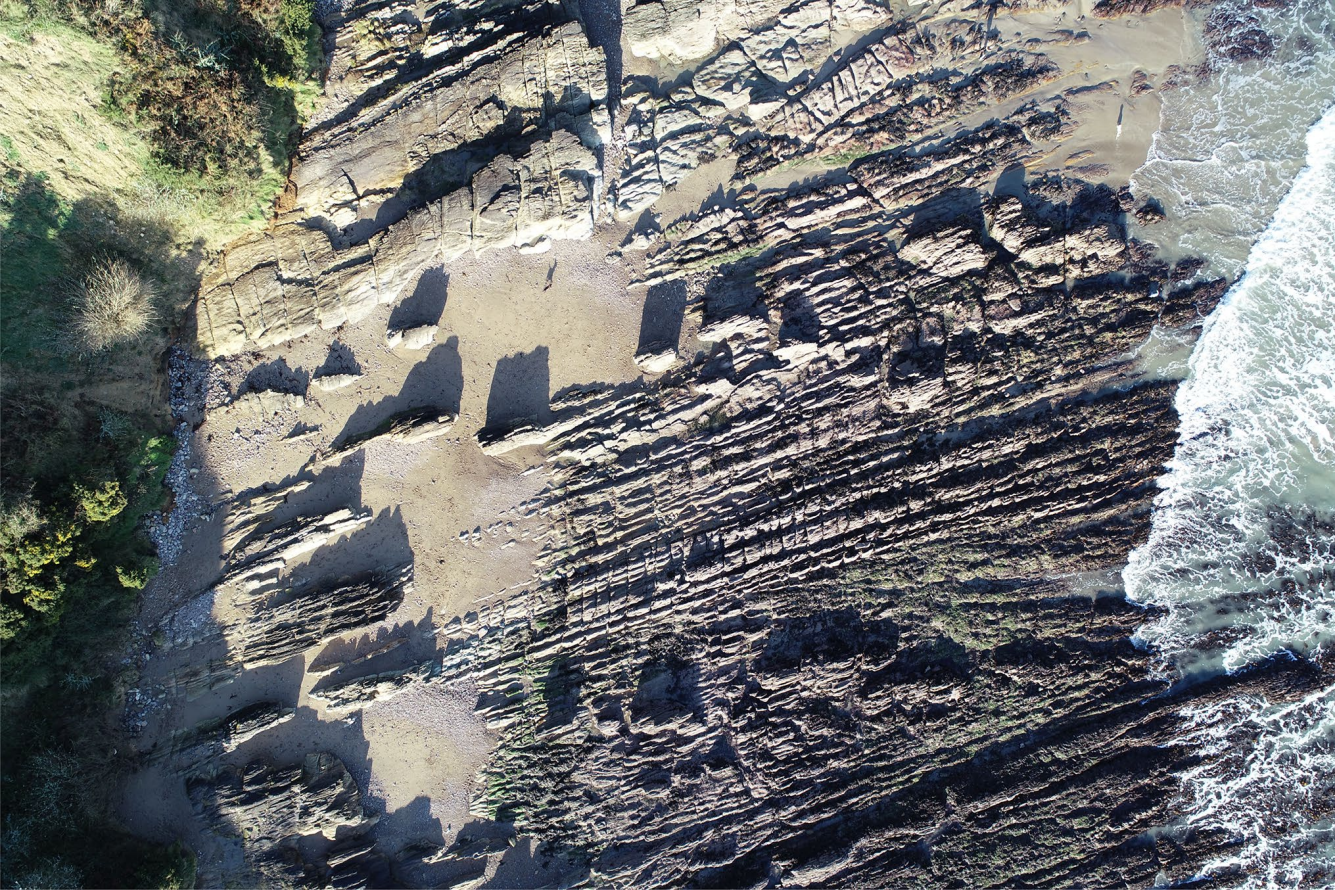
UAV imagery obtained as part of an ecological monitoring project investigating anthropogenic impacts on rocky shore ecology in Cork harbour (Ireland). [Source: Sarah Kandrot]

Mapping and identifying the geographic distribution of different habitats is a primary use of UAVs in the coastal environment (Fig. 2). Quantification of Blue Carbon stores has become a priority of many nations in response to the climate crisis, yet the global coverage of many sizable carbon stores, such as seagrass, remains largely unknown (Röhr et al. 2018; WEB 2014; Hastings et al. 2020). UAVs have been successfully used to map localised seagrass habitats, using multi-spectral, hyperspectral, and visible spectrum data (Duffy et al. 2018; Jeon et al. 2020). For example, Duffy et al. (2018) used a 3D Robotics Solo multi-rotor UAV with a 3D-printed sensor mount to collect hyperspectral imagery of *Zostera noltei* in Pembrokeshire, Wales. Blue Carbon habitats are increasingly being surveyed by UAVs, including mangrove forests (Ruwaimana et al. 2018; Wang et al. 2020; Warfield and Leon 2019) and saltmarshes (Green et al. 2020; Doughty and Cavanaugh 2019; Dai et al. 2020). By identifying the overall distribution of these habitats, estimates of the monetary value of carbon sequestration can be calculated, with Green et al. (2018) estimating the value of *Zostera marina* in the UK of between £2.6 and 5.3 million.

UAVs have also been used to identify nursery habitats for many rare and/or commercially important fish species. For example, Ventura et al. (2016) attached a GoPro to a quadcopter and used this imagery to undertake supervised classification of the RGB image bands, resulting in the creation of a fine-scale map of fish nurseries around Giglio Island in Italy. Such approaches to modelling fine-scale habitats of nurseries have been extended to identify the most suitable sites to undertake underwater censuses, which reduces the amount of time and resources for more targeted underwater surveys (Ventura et al. 2017). UAVs have also been used to map coral reefs (Silver 2019) with the quantification of change a key feature of these implementations, in part due to the ease and speed of mapping allowing for the potential regular monitoring of these habitats as UAVs become more ubiquitous.

UAVs are increasingly being used to limit interactions between people and animals in coastal areas. For example, Butcher et al. (2020) explored how UAVs can be used to manage conflict between swimmers and sharks, while quadcopters with live video streams have been deployed to increase the patrolling of the coastline to detect and enable real-time enforcement of illegal fishing (Howard 2016; Toonen and Bush 2020). The ability of larger UAVs to traverse up to 500 km over a 2-day period (Toonen and Bush 2020) enables UAVs to be highly effective in the management of this natural resource and is strongly tied to the enforcement and compliance of environmental law, which is further explored in the Environmental Law Enforcement and Compliance Section of this paper. UAVs have also been used to manage agriculture in terrestrial ecosystems, optimising agriculture operations, monitoring growth, and identifying the environmental impact of events such as flooding (Puri et al. 2017; Addo et al. 2018). Such information is key to the successful management of coastal communities to inform resilient planning opportunities, as well as to inform the provision of compensation to land managers. Further applications related to disaster assessment and management are discussed in the Disaster Assessment and Management Section of this paper.

From the 15 studies that provided sufficient detail, the average cost, ease of use, and maturity ratings were 3.07, 2.67, and 2.67, respectively, resulting in an overall accessibility rating of 8.41 out of 12, the 4th lowest of the nine applications. Many of the studies examined used simple consumer-grade UAVs and processing/analysis that required little or no training (e.g. Addo et al. 2018), with six having accessibility ratings of 9.0 or higher. However, some case studies were highly inaccessible, such as Silver (2019), which used a sensor priced at ~ €75,000 and required supercomputing for the processing.

Applications of UAVs for habitat mapping and biological and natural resource assessments are relevant to SDGs 14 and 15, and indicator 15.5.1 (Table 2).

**Table 2:** SDG goals and indicators to which drones can contribute data and information for habitat mapping and biological and natural resource assessment and management at the coast

SDG goal	Indicator	Relevance of drone technology
GOAL 14 Conserve and sustainably use the oceans, seas, and marine resources for sustainable development	None	Drone technologies can be used to document and map the distribution of marine and coastal resources (*e.g.* fisheries, seagrass meadows), providing policymakers, planners and managers with the evidence-base required for effective decision making and management
GOAL 15 Protect, restore, and promote sustainable use of terrestrial ecosystems, sustainably manage forests, combat desertification, and halt and reverse land degradation and halt biodiversity loss	Indicator 15.5.1 Red List Index	Drone technology can be used to document and map coastal biological resources (*e.g.* saltmarshes, mangrove forests) and to monitor changes in the distribution of threatened species

### Ecological Monitoring

Because they can provide high-resolution imagery in real time and at low cost, UAVs have emerged as a useful tool for ecological monitoring. For example, UAVs have been used to undertake bird censuses in both natural environments and urban environments to identify how populations are changing. In Fingal, Co. Dublin, Ireland, Roughan & O’Donovan (2018) used aerial imagery obtained from two UAVs (Yuneec H520 and the DJI Phantom 4 Pro +) to identify buildings on which herring gulls were nesting. They identified 451 nests in total and noted that birds favoured flat roofs or built their nests against chimney stacks. This work was commissioned in part to address the increasing conflict between humans and gulls in the region, but such UAV surveys are beginning to replace ground surveys of nesting sites for many remote coastal environments (e.g. Rush et al. 2018). Research has noted higher accuracy when using UAVs to conduct bird censuses compared with ground surveys in natural and often remote environments (e.g. cliff-tops). This is in part due to the increased ability of UAVs to identify camouflaged chicks; however, counts from UAV surveys may be skewed where birds flee their nests in response to an approaching UAV, exposing chicks to ground predators, thereby increasing predation rates (Brisson-Curadeau et al. 2017).

UAVs have also been successfully used to undertake aerial surveys of marine mammals. In Australia, Hodgson et al. (2013) undertook the first Australian UAV survey trial of dugongs. Using a ScanEagle UAV and digital SLR camera, they flew a 1.3km^2^ area capturing over 6000 images. They recorded over 600 sightings of dugongs, among other fauna including whales, dolphins, and turtles. They also noted a number of advantages of UAV surveys over traditional crewed surveys, such as an increase in the accuracy of detection and identification of species by minimising observer bias (proportion of animals visible to the observer but missed). Other similar works on the use of UAVs for collecting marine mammal count and density data include Ocean Research & Conservation Ireland (2020), Hensel, Wenclawski, and Layman (2018), Colefax et al. (2019), and Palomino Gonzalez (2019).

With many Environmental Impact Assessments now requiring aerial surveys as part of their remit, and conservation management plans requiring stock assessment to support funding allocations, UAVs may be seen a low-cost and efficient solution that can provide the data required for these activities. Such surveys are highly regarded due to their higher accuracy in counts; however, little is known regarding impacts on the behaviour of mammals in response to UAVs (Smith et al. 2016).

Monitoring the behaviour of marine taxa is becoming a prominent avenue for UAV research, particularly, due to the coupling of this technology with biologging techniques (Schofield et al. 2019). UAV footage can be monitored to quantify interactions between individuals. For example, Schofield et al. (2017) investigated whether departure of male sea turtles from breeding sites was driven by changes in the receptiveness of females or the probability of successful mating attempts, quantifying this through the sex ratio of all individuals within the footage. UAVs have also been used to monitor the health of species. For example, in Australia, UAVs were coupled with specialised equipment to capture the blow samples of 19 Eastern-Australian humpback whales in order to test for novel viruses (Geoghegan et al. 2018). They characterised the virome of the blow (or exhaled breath) and found six novel virus species. Such approaches are increasingly being used in Australia (see also Pirotta et al. 2017) and demonstrate the potential for such equipment to monitor the disease ecology of marine mammals.

Finally, from the eight studies that provided sufficient detail, the average cost, ease of use, and maturity ratings were 3.25 each, resulting in an overall accessibility rating of 9.75 out of 12, the joint 2nd highest of the nine applications. Six of the eight case studies have accessibility ratings of nine or higher, as many used simple visual observations from standard RGB cameras, a task can be performed with cheap UAVs and little training or expertise. Conversely, in one case study, Hodgson et al. (2013) employed insitu Pacific Pty Ltd to operate a ScanEagle, a UAV that costs ~ €500,000 and requires a high level of expertise to operate.

Applications of UAVs for ecological monitoring are relevant to SDG 15 and indicator 15.5.1 (Table 3).

**Table 3:** SDG goals, application, and indicators to which drones can contribute data and information for ecological monitoring in the coastal zone

SDG goal	Indicator	Relevance of drone technology
Goal 15 Protect, restore, and promote sustainable use of terrestrial ecosystems, sustainably manage forests, combat desertification, and halt and reverse land degradation and halt biodiversity loss	Indicator 15.5.1 Red List Index	Drone technology can be used to monitor the abundance, behaviour, and health of marine species, providing an evidence-base for more effective management activities

### Environmental Law Enforcement and Compliance

There are several examples of the use of UAV technology for environmental law enforcement and compliance. In the Philippines, Ezequiel et al. (2014) describe how UAV footage has been used to identify unregistered fish pens in one of the world’s most threatened lakes. In Ireland, eighteen local councils are using UAVs to visit known fly-tipping sites (“The View from above: Drones and Public Services” 2020). Waterford City and County Council are using footage from a DJI Phantom quadcopter in their investigations into fly-tipping and illegal dumping, which have led to several notices of fines being executed (Parker 2018). In Bantry Bay, the Irish Naval Service used a UAV to survey the extent of an accidental oil spill (O’Shea 2020). The footage can be used as part of an investigation into the incident. In the USA, UAVs are also being used at sea to support the enforcement of no-discharge zones, for example, where the release of bilge water is restricted (Alliance for Coastal Technologies 2018). Such footage can be used in court to increase the likelihood of prosecution.

UAVs have also been successfully employed in non-coastal settings for environmental law enforcement and compliance. Jiménez López and Mulero-Pázmány (2019) and Beaubien (2015) document how a conservation group is using fixed-wing UAVs to detect illegal mining and logging in the Peruvian Amazon. With their long range and ability to remain in the sky for long periods, the fixed-wing technology allows the group to quickly identify and investigate illegal activity. Nuwer (2017) describes how another conservation group is using thermal cameras, also mounted on fixed-wing UAVs, to combat poaching in Malawi’s Liwonde National Park. This allows them to detect illegal activity on the ground, even at night. Although not coastal, such applications could translate to ICZM.

Finally, from the four studies that provided sufficient detail, the average cost, ease of use, and maturity ratings were 3.25 each, resulting in an overall accessibility rating of 9.75 out of 12, the joint 2nd highest of the nine applications. All but one of the case studies used simple visual observations from the UAV footage to identify illegal activity, such as logging or dumping. The exception (Ezequiel et al. 2014) used a custom-made UAV and wrote some of their own code for processing tasks that may not be necessary nowadays with more modern equipment and software.

Applications of UAV technology for environmental law enforcement and compliance are relevant to SDGs 6, 14, and 15, and indicators 6.6.1, 14.4.1, and 14.6.1 (Table 4).

**Table 4:** SDG goals, targets, and indicators to which drones can contribute data and information in the area of environmental law enforcement and compliance at the coast

SDG goal	Indicator	Relevance of drone technology
GOAL 6 Ensure availability and sustainable management of water and sanitation for all	Indicator 6.6.1 Change in the extent of water-related ecosystems over time	Drones can help to identify and reduce illegal dumping and aquaculture activities, improving the quality of water-related ecosystems
GOAL 14 Conserve and sustainably use the oceans, seas, and marine resources for sustainable development	Indicator 14.4.1 Proportion of fish stocks within biologically sustainable levels	Monitoring illegal aquaculture practices can help to reduce destructive fishing practices
	Indicator 14.6.1 Degree of implementation of international instruments aiming to combat illegal, unreported, and unregulated fishing	Drones can help to improve the degree of implementation of international laws regulating unreported and unregulated fishing activities
GOAL 15 Protect, restore, and promote sustainable use of terrestrial ecosystems, sustainably manage forests, combat desertification, and halt and reverse land degradation and halt biodiversity loss	None	Drones can help identify poachers and illegal deforestation activities and bring perpetrators to justice

### Pollution Management

The use of UAVs to combat coastal and marine pollution has been documented in several instances. In Japan, Project Sea Unicorn (IKKAKU) promotes the development and commercialisation of innovative technologies that will reduce ocean debris (Leave a Nest Co. Ltd. 2020). Part of this research is the development of a global marine debris monitoring system that uses data from satellites and UAVs. These data will be used to predict the arrival of debris at the coast to make coastal clean-up operations more efficient, with a preliminary evaluation of such an application described by Watanabe et al. (2019). For example, a UAV swarm could be activated and sent to a particular area where abnormal trends are detected by satellite imagery to examine the area in more detail. Similar experiments have been conducted in Hawaii and Greece using various types of UAV platforms and sensors (Topouzelis et al. 2019, Papakonstantinou, and Garaba 2019; Alliance for Coastal Technologies 2018) and work is currently underway in Spain to test the technical and commercial potential of such techniques (Litterdrone 2020).

Several citizen science projects have also demonstrated how UAVs can help to address the marine litter problem (Fritz et al. 2019; Jiménez López and Mulero-Pázmány 2019; Campbell et al. 2019). Projects such as the Marine Litter UAVT encourage citizen scientists to undertake their own marine litter surveys using a standardised methodology (Kohler 2018). The images are being used to help train an object-detection system to automatically detect debris in UAV imagery. However, automatic detection is complicated because it is difficult to account for differences in physical conditions (e.g. weather, water turbidity, sun glint) between images taken in different locations or at different times.

Poor water quality due to nutrient pollution and wastewater discharge is another problem in some coastal and marine environments. Nutrient flows from industrial, urban, and agricultural activities can trigger harmful algal blooms (HABs), overgrowths of algae that can produce dangerous toxins. Alliance for Coastal Technologies (2018) describes how labs in Florida and Virginia, USA are exploring the use of UAVs for early detection and tracking of HABs using hyperspectral sensors. However, there are still many challenges to be overcome before this can become practical for coastal management activities, such as issues related to processing images of water, payload costs, and limitations, and a lack of standardised methods (Kislik, Dronova, and Kelly 2018; Martin et al. 2018).

From the four studies that provided sufficient detail, the average cost, ease of use, and maturity ratings were 1.75, 2.50, and 1.75, respectively, resulting in an overall accessibility rating of 6.00 out of 12, the lowest of the nine applications. These case studies tended to use more novel approaches than other applications, and also used more advanced UAVs and sensors, such as multi-spectral or thermal, and more advanced processing methods. This increased the costs, while lowering the ease of use and maturity ratings.

The applications of UAV technology to pollution management are of direct relevance to SDG targets 6 and 14 and indicators 6.3.1 and 6.3.2 (Table 5).

**Table 5:** SDG goals, targets, and indicators to which drones can contribute data and information in the area of coastal and marine pollution management

SDG goal	Indicator	Relevance of drone technology
GOAL 6 Ensure availability and sustainable management of water and sanitation for all	Indicator 6.3.1 Proportion of domestic and industrial wastewater flows safely treated	Multispectral, thermal, and hyperspectral sensors mounted on drone platforms can help to monitor water quality, although there are still many challenges to be overcome before this can become practical for coastal management activities
	Indicator 6.3.2 Proportion of bodies of water with good ambient water quality	
GOAL 14 Conserve and sustainably use the oceans, seas, and marine resources for sustainable development	None	Drones are an effective means of efficiently providing data and information on debris and nutrient pollution, assisting with targeted clean-up operations

### Digital Heritage Documentation, Preservation, and Conservation

The use of UAV technology for digital heritage documentation, preservation, and conservation is well documented, particularly, in relation to natural, built, cultural, and archaeological heritage sites. On behalf of English Heritage, a charity that manages hundreds of heritage sites in England, Historic England (2017) used a combination of UAV data, photogrammetry, and 3D printing to produce a 3D display model of Tintagel Island and Castle for an exhibition at a newly opened visitor's centre. The UAV survey was obtained using a visible camera mounted on fixed-wing and multi-rotor platforms to increase efficiency while ensuring maximum coverage of the site. Historic England processed the data using photogrammetry to create a 3D digital computer model, and then a reconstruction artist produced a physical 3D-scaled printed model of the island and a section of the adjoining mainland. In the visitor centre, an overhead projection system overlays the model with video showing the development of settlement and use of the island, enhanced by an audio soundscape. The project helps to educate the public and celebrate the natural and built heritage of the local area. Similar work was undertaken by Themistocleous et al. (2015) at the Byzantine church of Panagia Phorbiotissa in Asinou, Cyprus, a UNESCO World Heritage Site.

In Italy, Brumana et al. (2012) describe the use of visible oblique UAV imagery to support landscape heritage analysis and planning. They show how UAV images can be used to reconstruct panoramic views and simulate the visual impact of planned developments on the landscape, using examples from the Alpine foothills of Lombardy. The resulting imagery can be used to ensure that the proposed developments are in line with regulatory guidelines for provincial and municipal planning, such as those outlined in the European Landscape Convention. Also in Italy, Candigliota and Immordino (2013) showed how UAVs can be used to monitor and manage structural and architectural damage to build heritage sites following an earthquake. They used a visible camera mounted on helicopter and quadcopter platforms to survey a number of damaged historical buildings following an earthquake in Emilia-Romagna. The damage to the buildings meant that they were inaccessible, but they could be safely assessed from the high-resolution UAV imagery. This allowed the municipality to more efficiently plan recovery works. Similar work was also undertaken in Italy by Achille et al. (2015) and Trizzino et al. (2017), although rather than simply collecting 2D imagery, they used photogrammetry techniques to create 3D visualisations of damaged structures, which can be used by architects and engineers as a basis for reconstruction.

Many built heritage sites around the world are in poor condition, and this could present a public safety hazard. One such site is the abandoned Ashnott lead mine in North Lincolnshire, UK. Historic England (2017) surveyed the site using a fixed-wing UAV to highlight places where the collapse of old, poorly sealed shafts presented a danger to livestock and hill-walkers. The surveys were then used to plan the installation of new fences, intended to safeguard the remains.

Many of the world’s coastal heritage sites have been submerged or are under imminent threat of submergence because of changing sea-levels. In Greece, several submerged ancient harbours have been discovered along the coast of Lesbos Island, in the Aegean Sea (Harff, Bailey, and Lüth 2016). Papakonstantinou et al. (2019) explored how UAVs can be used to map such sites. They used visible UAV imagery and photogrammetry techniques to create detailed 3D models of two sites at Mytilene and Eresos. This enabled the discovery of new hidden structures. Similar studies have been undertaken in Cyprus (Skarlatos and Savvidou 2015), Russia (Nicu et al. 2019), and Ireland (CHERISH 2020) with authors noting technical difficulties in creating accurate orthomosaics of submerged sites, but with the potential for such information to be used for future management plans and conservation strategies.

Several others document the use of UAV technologies for archaeological heritage investigations and conservation in coastal settings. In Australia, Kurpiel et al. (2018) used visible imagery and photogrammetry to record the presence of Aboriginal cultural heritage at a coastal dune site prior to the imminent agricultural development of the landscape. The pending development meant that the physical and topographical context in which artefacts were found could be lost forever. The digital recording will allow archaeologists to continue to investigate the site (to some extent), even after development has occurred. In Canada, researchers at the Hakai Institute are using UAV technology to create detailed maps of ancient clam gardens created by First Nations (Alliance for Coastal Technologies 2018). Many such sites have been destroyed by storms, tectonic activity, or historic logging activities (Holmes 2016). By digitally recording the remains of the surviving sites, a record of their existence remains. SDG goal 11 specifically emphasises the protection and safeguarding of the world’s natural and cultural heritage sites. The applications outlined above demonstrate how UAV technology can help to contribute to this endeavour.

From the nine studies that provided sufficient detail, the average cost, ease of use, and maturity ratings were 3.11, 2.89, and 3.11, respectively, resulting in an overall accessibility rating of 9.11 out of 12, ranked 5th of the nine applications. Most of the case studies use Structure from Motion photogrammetry, which can lower the ease of use a little, but it is a commonly used form of UAV-based analysis, meaning the maturity was high. Relatively cheap off-the-shelf UAVs were used most frequently, helping to keep the costs down.

The applications of UAV technology to for digital heritage documentation, preservation, and conservation are of direct relevance to SDG 11 and indicator 11.5.2 (Table 6).

**Table 6:** SDG goals, targets, and indicators to which drones can contribute data and information in the area of digital heritage preservation, documentation, and conservation at the coast

SDG goal	Indicator	Relevance of drone technology
GOAL 11 Make cities and human settlements inclusive, safe, resilient, and sustainable	None	Drone technologies can be used to document, digitally preserve, and manage cultural and natural heritage sites
	Indicator 11.5.2 Direct economic loss in relation to global GDP, damage to critical infrastructure and number of disruptions to basic services, attributed to disasters*	Drone technologies can be used to (safely) help quantify the economic loss of damaged or destroyed cultural heritage sites in the event of a disaster (e.g. following an earthquake)

*A metric that contributes to this indicator is ‘Direct economic loss to cultural heritage damaged or destroyed attributed to disasters’

### Emergency Response

UAVs can be very effective tools in supporting search and rescue activities (Jiménez López and Mulero-Pázmány 2019; Rajabifard 2019; Burke et al. 2019). UAV platforms equipped with visible or thermal sensors can be used to search large, potentially difficult-to-access or inaccessible areas and detect people in distress on the ground or at sea. Thermal sensors are particularly suited to such activities because warm objects, such as people or animals, stand out against the relatively cooler background in the false colour images. This makes them easier to detect than from visible imagery or from the view of rescuers aboard an airplane or helicopter. This is critical in time-sensitive situations, where people’s lives may be at risk. Thermal cameras have the added advantage of being able to detect objects both in the day or at night. Manual detection of humans from the imagery or video footage, though, can be a difficult task, especially over a large search area. Artificial intelligence (AI) algorithms are now under development to automate the detection of people, both from visible and thermal UAV imagery, for search and rescue operations. These, however, are still in the very early stages of development and not yet practical for real-life operations (Burke et al. 2019; Lomonaco et al. 2018). Nevertheless, Lomonaco et al. (2018) suggest such AI-equipped platforms could eventually be used to power ‘intelligent UAV swarms’ for search and rescue operations at sea, for example, in the context of the migrant crisis in the Mediterranean Sea. Other examples of potential UAV uses in search and rescue operations include use of UAV imagery to gather information on the ground state, use of UAVs as communications relay points, and use of UAVs to deliver life-saving equipment, such as life vests, to people in distress (Bäckman et al. 2018; Seguin et al. 2018; Burke et al. 2019). In 2018, the leading UAV manufacturer DJI published a report documenting real-world examples of UAV use in search and rescue operations. Between May 2017 and April 2018, they counted at least 65 people who were rescued from peril by use of a UAV (DJI Technology Inc. 2018).

From the four studies that provided sufficient detail, the average cost, ease of use, and maturity ratings were 2.50, 3.00, and 2.75, respectively, resulting in an overall accessibility rating of 8.25 out of 12, the 3rd lowest of the nine applications. The accessibility of UAV for emergency response varied significantly based on the task. For example, Bäckman et al. (2018) used a standard UAV with a commercially available attachment to drop off floatation devices, giving it a high accessibility rating of 11. Conversely, Burke et al. (2019) used a custom-built UAV with an expensive thermal camera and machine learning for automated detection, lowering the accessibility to 6.0.

Such applications of UAV technology to emergency response are of direct relevance to SDGs 1, 1, 11, and 13 and indicators 1.5.1, 10.7.2, 11.5.1, and 13.1.1 (Table 7).

**Table 7:** SDG goals, targets, and indicators to which drones can contribute data and information for emergency response at the coast

SDG Goal	Indicator	Relevance of drone technology
GOAL 1 End poverty in all its forms everywhere	Indicator 1.5.1 Number of deaths, missing persons and directly affected persons attributed to disasters per 100,000 population	Drones can help to reduce the number of deaths, missing persons, and directly affected persons attributed to disasters by quickly and cheaply providing rescuers with the information they need to find people in distress
GOAL 10 Reduce inequality within and among countries	Indicator 10.7.2 Number of countries with migration policies that facilitate orderly, safe, regular, and responsible migration and mobility of people	Drones can be used to support search and rescue of migrants, refugees, and internally displaced persons at sea, for example, those who find themselves in difficulty crossing the Mediterranean Sea
GOAL 11 Make cities and human settlements inclusive, safe, resilient, and sustainable	Indicator 11.5.1 Number of deaths, missing persons and directly affected persons attributed to disasters per 100,000 population	Drones can help to reduce the number of deaths, missing persons, and directly affected persons attributed to disasters by quickly and cheaply providing rescuers with the information they need to find people in distress
GOAL 13 Take urgent action to combat climate change and its impacts	Indicator 13.1.1 Number of deaths, missing persons and directly affected persons attributed to disasters per 100,000 population	

### Disaster Assessment and Management

Assessment of the damages from coastal disasters (e.g. storms, earthquakes, oil spills) is important for planning recovery activities, assessing insurance claims, and increasing the capacity of coastal communities to respond to future events. UAVs are commonly used to support these activities. For example, the use of UAVs to assess post-storm coastal erosion and damage to built infrastructure is well documented (M.J. Starek, Gingras, and Jeffress 2019a, b; Alliance for Coastal Technologies 2018; Scarelli et al. 2017; Duo et al. 2018; Laporte-Fauret et al. 2019). On the southwest coast of France, Laporte-Fauret et al. (2019) demonstrated how a consumer-grade quadcopter and photogrammetry techniques could be used to monitor post-storm beach and dune erosion. They showed how high-resolution digital surface models, which can be used to measure morphological and sediment volume changes after a storm or winter storm season, could be created from the visible imagery. The quality of their models were found to be sufficient for coastal monitoring, and the authors highlight the fact that UAV data can be obtained cheaper, faster, and more efficiently than aerial LiDAR or traditional ground survey techniques. Similar work has been undertaken in the USA for hurricane and storm impact assessment (Starek et al. 2019a, b; Scarelli et al. 2017; Alliance for Coastal Technologies 2018; Duo et al. 2018). Authors continually highlight the usefulness of the low-cost technique for evaluating the effectiveness of these different shoreline protection measures, which can ultimately support local authorities in the development and implementation of ICZM plans.

Quick access to information is essential to efficiently plan for disaster management. In Italy, Trizzino et al. (2017) used UAV surveys to detect hazards posed by erosion of rocky cliffs, known locally as ‘falesie’, by using visible UAV images to create 3D models of the cliffs, which were compared with LiDAR surveys obtained previously, allowing them to map the fractured fronts of the cliffs in detail along a 1-km stretch of coast in Lecce, Southern Italy. Feng et al. (2015) and Popescu et al. (2017) demonstrate successful use cases of how UAV data can evaluate hazards posed by flooding in urban areas, while Ezequiel et al. (2014) and Popescu et al. (2017) illustrate the use of visible UAV imagery to support post-typhoon and earthquake responses, respectively. Such research highlights that this information can be used by local authorities to distribute funds and by insurance companies to determine payments.

From the seven studies that provided sufficient detail, the average cost, ease of use, and maturity ratings were 2.29, 2.43, and 2.86, respectively, resulting in an overall accessibility rating of 7.57 out of 12, the 2nd lowest of the nine applications. Some case studies used cheap, standard UAVs, and established processing methods (Duo et al. 2018; Laporte-Fauret et al. 2019) resulting in a high accessibility rating. Several others used custom built or expensive UAVs (Feng et al. 2015; Popescu et al. 2017) and or complex processing methods (Feng et al. 2015; Popescu et al. 2017; Ezequiel et al. 2014), which reduced their overall accessibility.

The applications of UAV technology to disaster assessment and management are directly relevant to SDGs 1, 11, and 13 and indicators 1.5.2, 11.5.2, and 13.1.1 (Table 8).

**Table 8:** SDG goals, targets, and indicators to which drones can contribute data and information in the area of disaster assessment and management at the coast

SDG goal	Indicator	Relevance of drone technology
GOAL 1 End poverty in all its forms everywhere	Indicator 1.5.1 Number of deaths, missing persons, and directly affected persons attributed to disasters per 100,000 population*	Drones can help to quantify damage to dwellings, built infrastructure, agricultural land, and other assets following a disaster, which can also help with estimation of economic losses
	Indicator 1.5.2 Direct economic loss attributed to disasters in relation to global gross domestic product (GDP)	
GOAL 11 Make cities and human settlements inclusive, safe, resilient, and sustainable	Indicator 11.5.1 Number of deaths, missing persons, and directly affected persons attributed to disasters per 100,000 population*	
	Indicator 11.5.2 Direct economic loss in relation to global GDP, damage to critical infrastructure, and number of disruptions to basic services, attributed to disasters**	
GOAL 13 Take urgent action to combat climate change and its impacts	Indicator 13.1.1 Number of deaths, missing persons, and directly affected persons attributed to disasters per 100,000 population***	Drones can help to quantify damage to dwellings following climate-related disasters

### Planning and Development

Effective planning is a key tenet of ICZM and is essential to achieving the SDGs (Burbridge 1999; West 2019). Several studies have highlighted how UAVs can support planning authorities in the development and implementation of ICZM plans. For example, Scarelli et al. (2016) demonstrated how maps generated from UAV surveys could be used to identify anthropogenic pressures on a beach/dune system in Brazil. This information could be used to secure funding for local ICZM, especially where local authorities have limited funds/resources to acquire high-quality mapping and monitoring data. Similar work by Scarelli et al. (2017) (described in the Disaster Assessment and Management Section of this paper) demonstrates how UAV surveys can be used to evaluate the effectiveness of different coastal management interventions, thus supporting local authorities in the development and implementation of evidence-based ICZM plans. Sabetien et al. (2019) showed how UAV imagery could be used to guide seaweed cultivation planning in the Solomon Islands. They developed a decision support system (DSS) using a GIS technique called spatial multiple criteria analysis (MCA), which allowed them to identify suitable locations for seaweed farming based on multiple, conflicting criteria. Among those criteria were the distribution of the resource and the extent of existing farms, which were mapped from UAV imagery. The imagery is further intended to provide useful site information for future farm development, planning, and monitoring and will help support the sustainable development of seaweed farming in the Solomon Islands.

Work by Brumana et al. (2012), described in the Digital Heritage Documentation, Preservation, and Conservation Section of this paper, demonstrates how UAV imagery can be used to support landscape planning by helping to visualise how different development scenarios would impact on waterfront views. Since the images can be easily shared via web-based geoportals, Brumana et al. (2012) argue that this can enhance public participation in the planning process. Ezequiel et al. (2014) used UAV imagery to map land-use development trajectories around the Seven Lakes of San Pablo in the Philippines. They contend that such maps can be used to support long-term infrastructure and zoning plans. Similarly, Gallagher and Lawrence (2016) highlight the potential for UAV imagery to support sustainable land-use planning in urban environments. For example, high-resolution maps can help to identify and plan the potential re-use of abandoned urban areas within cities. They can also help with planning and management of green and open spaces—aiding in inventory studies of the health of urban trees to determine stresses, evidence of infestations, disease, the need for addition of new trees, etc. UAVs can also support marine spatial planning. Themistocleous et al. (2019) used UAV imagery in combination with other geospatial data to identify sea and land activities around the ports of Cyprus. This information is essential for the implementation of integrated marine plans in compliance with the European Marine Spatial Planning Directive (2014/89/EU).

Finally, from the 4 studies that provided sufficient detail, the average cost, ease of use, and maturity ratings were 3.50, 2.75, and 3.00, respectively, resulting in an overall accessibility rating of 9.25 out of 12, the 4th highest of the nine applications. The case studies mainly used cheap UAVs, which caused a high cost rating, but the custom-built nature of the UAV and the processing methods used by Ezequiel et al. (2014) lowered the ease of use and maturity ratings.

Applications of UAV technology for planning and development are relevant to SDGs 14 and 16 (Table 9).

**Table 9:** SDG goals, targets, and indicators to which drones can contribute data and information in relation to planning and development at the coast

SDG Goal	Indicator	Relevance of drone technology
GOAL 14 Conserve and sustainably use the oceans, seas, and marine resources for sustainable development	None	Drone technology can be used to support marine spatial planning, which will aid in the sustainable management and protection of marine and coastal ecosystems. For example, drones can be used to monitor development activities, such as seaweed farming, which can contribute to the implementation of ICZM and marine spatial plans
GOAL 16 Promote peaceful and inclusive societies for sustainable development, provide access to justice for all and build effective, accountable, and inclusive institutions at all levels	None	Drone imagery can be used to enhance public participation in the planning process

### Tourism

UAV imagery and videos are well placed to promote tourism and to facilitate ‘virtual tourism’—the experience of visiting a place through digital media without physically being present in that place (Kitonsa and Kruglikov 2018). Several companies with expertise in UAV photography/videography and virtual reality (VR) imaging technology are now offering to create customised, sometimes interactive, virtual tours for businesses in the tourism sector (3deep 2020; Virtual Reality Tour Guys 2020; e.g. Drone View 2020). The tours can be simply made up of images or video footage or they can be more complex, for example, offering visitors the chance to explore an area in 3D with supplementary educational information embedded in the tour (Mirk and Hlavacs 2014). The tours can be delivered via the web for viewing on a computer or mobile device or even using VR goggles for a more immersive experience (Rutkin 2015; DHLGH 2017).

Besides being used for marketing and promotion, UAV imagery and footage can help to limit visitors to certain areas threatened by excessive human activity, thereby contributing to more sustainable tourism practices. For example, the high number of visitors to the South Ari atoll Marine Protected Area (SAMPA) in the Maldives is posing a threat to resident marine species (Femmami 2019). Interactions with an increasing number of boats are causing injury to a high proportion of local whale sharks (Allen 2018). In addition, overcrowding threatens the safety of swimmers and negatively affects visitors’ satisfaction. Femmami (2019) argues that UAVs can be used to help alleviate these problems. For example, UAVs can be used by rangers to perform aerial surveillance operations, giving them the information they need to disperse tour operators among a greater number of sharks, thereby limiting interactions with boats as well as the potential danger to swimmers.

‘Virtual tourism’ has perhaps never been more relevant than in the time of coronavirus (Katanich 2020). In Ireland, Virtual Visit Tours uses a combination of ground and UAV imagery to create 360 degree immersive media tours of popular tourist destinations (Virtual Visit Tours 2020). The tours have been featured as destinations for virtual visitors who cannot physically travel to the island or site as a result of travel restrictions or temporary closure due to Covid-19 (Romano 2020). Other examples of virtual tourism include a virtual UAV tour of Pompeii (Osanna 2020) and tours of (mostly UK-based) tourist destinations created by 3deep (3deep 2020). Traditionally, virtual tourism was seen as a way to promote a location to attract visitors, usually commissioned by hotels, tourist sites, and other businesses in the tourism industry, but in the last few months it has emerged as an entertaining and educational way of visiting tourist destinations that are temporarily off-limits. Such tours can be monetised, for example, by generating ad revenue from YouTube and other social media sites. The revenues can potentially be used by local coastal communities to promote the sustainable development of the tourism sector.

From the four studies that provided sufficient detail, the average cost, ease of use, and maturity ratings were 3.50, 3.25, and 3.25, respectively, resulting in an overall accessibility rating of 10.00 out of 12, the highest of the nine applications. Most of the case studies used simple visual media, such as videos or photos, with little or no additional processing, making them highly accessible. Mirk and Hlavacs (2014) attempted to allow remote users to control a UAV to explore the landscape in real-time, adding considerable complexity to the task and reducing the ease of use and maturity.

Tourism applications of UAV technology are relevant to SDGs 8, 11, and 12 and indicators 8.9.1 and 12.b.1 (Table 10).

**Table 10:** SDG goals, targets, and indicators to which drones can contribute data and information in relation to tourism at the coast

SDG Goal	Indicator	Relevance of drone technology
GOAL 8 Promote sustained, inclusive, and sustainable economic growth, full and productive employment, and decent work for all	Indicator 8.9.1 Tourism directs GDP as a proportion of total GDP and in growth rate	Drone imagery/footage can be used in ‘virtual tourism’ products, which can be monetised, providing a source of income for local businesses, and the revenues can potentially be used by local coastal communities to promote the sustainable development of the tourism sector
GOAL 11 Make cities and human settlements inclusive, safe, resilient, and sustainable	None	Drone technology can help to limit the impacts of tourism on cultural and natural heritage sites at the coast, for example, by helping to deliver ‘virtual tourism’ as an alternative to physical visits to threatened sites
GOAL 12 Ensure sustainable consumption and production patterns	Indicator 12.b.1 Number of sustainable tourism strategies or policies and implemented action plans with agreed monitoring and evaluation tools	Drone imagery/footage can be used in ‘virtual tourism’ products, which can create jobs and promote local culture and products

## UAVs & SDGs: Reflections, Challenges, and Future Directions

With the advent of low-cost consumer-grade UAVs, the technology has become ubiquitous among many organisations and institutions, including many local government agencies in North America, Oceania, and Europe (Miller 2018; council.ie 2019; Gee 2019). Among its many uses, the technology is well placed to support ICZM activities because it can deliver relevant data and information that can contribute to evidence-based decision making. Many local authorities struggle with the implementation of ICZM, due in part to limited access to resources and funding (Cummins et al. 2004). The low operating cost, high level of automation, and high quality of survey data from modern UAV technology can help support local authorities in their efforts to implement ICZM (Scarelli et al. 2017). As part of the 2030 Agenda for Sustainable Development (UN General Assembly 2015), the SDGs are composed of 17 goals that address global challenges. For each goal, several targets have been defined, and for each target, there are a number of indicators and metrics for measuring progress towards those targets, and, ultimately, the overall goal. Unfortunately, it is difficult to quantify many of those metrics, but there are many areas where UAV technology can help, and we highlight these specific avenues in Tables 2–10.

**Fig. 3:**
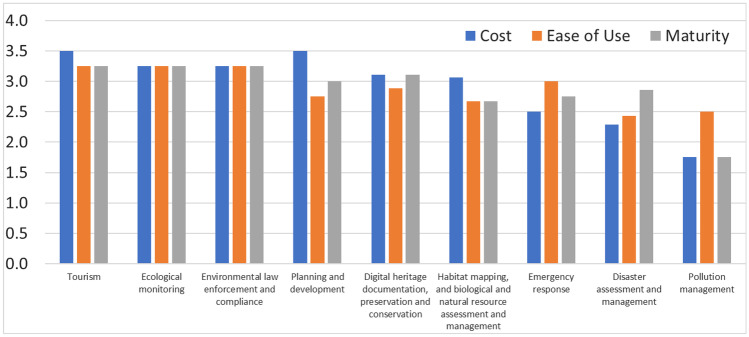
Accessibility scores for cost, ease of use, and maturity of the nine areas in which UAV technologies can support ICZM and the UN SDGs at the coast

Overall, we identified ten (out of the 17) SDGs to which UAVs can contribute data and information (Tables 2–10). For the most part, these areas recorded high (> 2.5 out 4) accessibility criteria for all the applications, with the exception of pollution management and disaster management (Fig. 3). As stated, the pollution management case studies tended to use more novel approaches than other applications, and used more advanced UAVs and sensors, such as multi-spectral or thermal, and more advanced processing methods. This increased the costs, while lowering the ease of use and maturity ratings. However, the majority of examples provided within this review did not require such advanced accessibility levels, and while no means exhaustive of all possible applications, they demonstrate that such technology is available with relatively high accessibility relating to cost, ease of use, and maturity which should support a wide array of stakeholders to address several sustainability challenges set out by the SDGs.

Finally, while many new and exciting coastal applications have emerged out of rapid advances in UAV technology, there remain a number of practical and technological challenges that must be addressed, including automated image processing, establishing a set of best practices, public participation, equipment limitations, limited experience, compliance with policy, and privacy regulations.

### Automated Image Processing

There is still a high degree of manual work and skill involved in UAV image classification for coastal applications (*e.g.* seaweed resource assessments, mapping of bacterial pathogens for monitoring coastal bathing water quality, automated detection of people from UAV imagery for search and rescue applications). These applications often resulted in the lower scores of accessibilities (Table 1, Fig. 3), suggesting that such techniques are perhaps the primary limiting factor for certain ICZM and SDG considerations. In addition, the subjectivity of some approaches makes them difficult to repeat, but accounting for changes in physical conditions, such as seasonality, weather, water turbidity, and sun glint, complicates automated classification (Joyce et al. 2019). The application of machine learning and other AI algorithms to UAV data processing is emerging as an active research area (e.g. Gonçalves and Henriques 2015; Pashaei et al. 2020). However, further work addressing how variable conditions can be dealt with is required before accurate, automated, user-friendly tools can be developed for practical coastal applications (Burke et al. 2019; Lomonaco et al. 2018).

### Best Practice

Surveys in environmentally sensitive areas require consideration of how to minimise or avoid potential negative impacts on wildlife, such as birds. Little is known regarding impacts on animal behaviour response to UAVs, and there is a need for further research and recommendations for best practice, for example, in relation to various species of birds and marine mammals (Brisson-Curadeau et al. 2017).

### Public Participation

As the cost of UAV platforms has reduced, usage of the technology has expanded to hobbyists and citizen scientists. Several citizen science projects have recently emerged, including the Marine Litter UAVT (Kohler 2018), Open Reef (ESRI 2020), and Citizen Science Coastal Monitoring (Deakin Marine Mapping 2020). However, within the citizen science community there is a lack of standardised methods for collecting UAV data (Sunyer et al. 2019). Best practice guidance should be established and promoted to citizen science groups.

### Equipment Limitations

The technological capabilities of UAV platforms and sensors are evolving rapidly. However, the practical usage of the current generation of UAV systems is constrained by the specifications of the equipment. For example, battery power limits flight time, meaning a battery-powered UAV may need to take off and land multiple times to change the batteries. This may not be practical for some applications. Research is currently underway on how to improve the endurance of UAVs (Jung, Jo, and Kim 2019; Castro et al. 2020). Methodologies for coastal applications should take into account such practical considerations, and this review has tried to provide an objective basis for such accessibility considerations.

### Limited Experience

In 2018, a workshop was held in Maine, USA on the practical uses of UAVs to address management problems in coastal zones. The workshop summary report concluded “[coastal] managers are eager to use UAVs, but how to use them is not always well understood” (Alliance for Coastal Technologies 2018, p. 3). In addition, a recent study involving 350 local authorities in the UK indicated that despite the increasing adoption of UAV technology by local councils, many do not have appropriate policies in place to ensure that they are compliant with UAV regulations (Gee 2019). Such policies are important to limit safety and/or legal issues. Subsequently, there is a need for best practice guidance on the use of UAV technology for coastal practitioners, as well raising awareness of the existence of such policies. 

### Compliance with Local Regulatory Frameworks

A key practical consideration for the applicability of UAV technology is the local regulatory framework in relation to the safe use of UAVs. This can potentially limit some applications. For example, most countries require that UAVs be flown within the operator’s visual line of sight (VLOS). In other words, the UAV must be visible in the sky at all times. If a very large survey is to be undertaken, this could mean taking off and landing at multiple locations, limiting the efficiency of the technology. There is considerable diversity in UAV legislation between countries (Tsiamis et al. 2019), although national rules in the European Union were  replaced by a common EU legislation in 2020 to facilitate cross-border operations. As legislation changes to keep up with technological advances, it is important for UAV operators/coastal practitioners to keep themselves informed, not only to decide if an application is feasible, but also to avoid fines, injuries, or damage to property.

### Privacy and Data Protection

Legislation in relation to UAVs includes regulations on privacy and data protection. For example, in the EU, non-recreational UAV operations must comply with the Data Protection Directive (Directive 95/46/EC) on the protection of individuals with regard to the processing of personal data and on the free movement of such data. Personal data that can inadvertently be captured by UAVs include facial images or car registration plates. UAV operators have certain obligations to ensure such data are handled appropriately and in line with national and international laws. This is especially important in coastal environments, for example, on popular beaches.

## Conclusion

Effective ICZM practices, as well as progress towards the SDGs, necessitates accurate data about the coast (Politi et al. 2019). UAVs can deliver such data on-demand over larger areas than are practicable using ground surveys and at a higher spatial and temporal resolution than EO data. These attributes are particularly useful for a range of coastal applications, such as monitoring beach morphology change, habitat mapping and ecological monitoring, and several other applications described in this paper. Furthermore, the data obtained from UAV surveys can be used to support the delivery of and measure progress towards ten of the UN SDGs at the coast. Some of the key requirements going forward include further development of automated image processing algorithms that consider variable conditions, development of best practice methodologies for coastal practitioners and citizen scientists, and awareness raising in relation to best practice and compliance with local legislation.

## Supplementary Information

Below is the link to the electronic supplementary material.

Supplementary file1 (XLSX 54 kb)
